# Effects of the Zishen Yutai Pill compared with placebo on pregnancy outcomes among women in a fresh embryo transfer cycle: a *Post Hoc* subgroup analysis of a randomized controlled trial

**DOI:** 10.3389/fendo.2023.1196636

**Published:** 2023-11-21

**Authors:** Xiaoli Chen, Yu Li, Jiewen Zhou, Xuemei Wei, Na Ning, Qiuling Huang, Xiufei Pang, Dongzi Yang

**Affiliations:** ^1^ Center for Reproductive Medicine, Sun Yat-Sen Memorial Hospital of Sun Yat-Sen University, Guangzhou, China; ^2^ The First Clinical College, Guangzhou University of Chinese Medicine, Guangzhou, China; ^3^ Guangdong Development Engineering Laboratory of Southern Chinese Herbal Drugs, Guangzhou, China

**Keywords:** Zishen Yutai pill, *in vitro* ferilizaion, embyro transfer, subgroup analysis, tradtional chinese medicine

## Abstract

**Objective:**

To assess whether the administration of Zishen Yutai Pill (ZYP) could improve the pregnancy outcomes in different subgroups of women undergoing fresh embryo transfer cycles.

**Materials and methods:**

This is a *post hoc* analysis of a large scale, placebo-controlled, double blind, randomized clinical trial (RCT) regarding the use of ZYP during assisted reproductive technology (ART) treatment. The RCT was conducted at 19 *in vitro* fertilization (IVF) centers between April 2014 and June 2017. A total of 2265 women undergoing fresh embryo transfer cycles were randomly assigned in a 1:1 ratio to receive ZYP (n = 1131) or placebo (n = 1134). *Post hoc* logistic regression analyses were applied in this study to examine the between-group differences of ZYP and placebo on clinical pregnancy rate among different subgroups. Detailed analyses, both in intention-to-treat (ITT) and per-protocol population, were also conducted in specific subgroups with regards to rates of implantation, biochemical pregnancy, clinical pregnancy, live birth, pregnancy loss, as well as other neonatal indices.

**Results:**

ZYP showed a significantly higher clinical pregnancy rates than placebo in the ITT population. Detailed subgroup analyses were conducted in subgroup in advanced maternal age (AMA, ≥ 35 years old) and overweight/obese patients (BMI > 24), due to the clinical importance and statistical results. In these subgroups, baseline characteristics were similar between two arms (all P > 0.05). Significantly elevated clinical pregnancy rates were observed in ZYP cohort (both P < 0.05) compared with the placebo group. Results also showed that ZYP treatment resulted in significantly higher rates of implantation, biochemical pregnancy in AMA or overweight/obese patients in ITT analysis (all P < 0.05).

**Conclusions:**

The current *post hoc* subgroup analysis suggested that AMA and overweight/obese women could experience clinical benefits when treated with ZYP in their fresh embryo transfer cycles. The study provides references for the use of ZYP in ART practices. However, further studies in specific subgroups should be examined in more rigorous clinical trial settings.

**Clinical trial registration:**

Chictr.org.cn, ChictrTRC-14004494.

## Introduction

Female infertility has become an urgent problem affecting the well-being of couples at their reproductive age. The number of women actively seeking for infertility treatment is now drastically increasing in the past few decades (1). It is estimated that lifetime prevalence of infertility is estimated to be 17.5%, and 48 million couples live with infertility globally (2, 3). *In vitro* fertilization and embryo transfer (IVF-ET) is widely used for dealing with infertility and has resulted in millions of births worldwide (4–6). Over the past decades, significant advances have been made to improve IVF-ET outcomes. However, pregnancy rates are still in a relatively low level due to poor oocyte quality, embryo implantation disorder, poor endometrial receptivity and other problems caused by controlled ovarian hyperstimulation (COH) (7–9). Nowadays, several treatment options are applied in the improvement of pregnancy outcomes during assisted reproductive technology (ART) treatment, such as dehydroepiandrosterone (DHEA), growth hormone, coenzyme Q10, acupuncture and traditional Chinese Medicine (TCM) (10–13). In the field of infertility therapies, TCM has attracted more and more attention. Clinical practice has shown that TCM exhibits complementary effects during ART, improving pregnancy outcomes of infertile women. Emerging evidences have shown that TCM exerts therapeutic effects through various mechanisms, including improvement of oocyte and embryo quality, amelioration of endometrial receptivity, regulation of sexual hormones, etc. (14–16).

Zishen Yutai Pill (ZYP) is one of the representative TCM preparations used in IVF-ET. ZYP consists of 15 natural medicines, including Cuscutae Semen, Ginseng Radix et Rhizoma, Dipsaci Radix, Taxilli Herba, Eucommiae Cortex, Morindae Officinalis Radix, Cervi Cornu Degelatinatum, Codonopsis Radix, Atractylodis Macrocephalae Rhizoma, Asini Corii Colla, Lycii Fructus, Rehmanniae Radix Praeparata, Polygoni Multiflori Radix Praeparata, Artemisiae Argyi Folium, Amomi Fructus (14). Recently, we conducted a multicenter, large-scale randomized clinical trial (RCT), showing that ZYP administration could increase live birth rates after fresh embryo IVF cycles when compared with placebo (17). Pharmacological studies demonstrated that ZYP could ameliorate advanced endometrial maturation through the upregulation of HOXA10, and may improve oocyte function via upregulating TGF-β (18, 19). Meanwhile, a metabonomic study also suggested that metabolites involved in various pathways and biological processes that may related to the improvement of endometrium receptivity and oocyte quality after ZYP administration (20). A virtual screening further indicated that regulation of neuroactive ligand-receptor interaction, steroid hormone biosynthesis, and ovarian steroidogenesis might be the potential therapeutic mechanism of ZYP in treating premature ovarian failure (POF) (21). Clinically, ZYP used in the luteal phase was reported to improve pregnancy outcomes among patients undergoing IVF-ET (22).

It is well-documented that maternal factors, such as advanced age, overweight and obesity, have adverse impacts on the pregnancy outcomes (23–25). For example, age is identified as an independent risk factor for female fecundity and their pregnancy outcomes. With more and more women postpone their marriage and childbearing nowadays, the proportion of advanced maternal aged mothers was considerably increased (26–28). Another risk factor during pregnancy is the high body mass. Obesity or overweight could lead to poor pregnancy outcomes in IVF (29). Several guidelines and expert consensus from China and other countries have been developed to address the ART strategies for infertile women with advanced age and overweight/obesity (30–35). Whether taking ZYP would provide higher benefits in these subpopulations has not been assessed. To this end, we performed an exploratory *post hoc* analysis using the data from an RCT to compare the between-group differences of ZYP and placebo across different subgroups.

## Materials and methods

### Study design and participants

The study design has been reported in detail (17). Registration was made on April 13, 2014 (Chictr.org.cn, Chictr-TRC-14004494). Approval by the Ethics Review Committee was sought at each participating site and written informed consent from all subjects were obtained. Following is a brief overview of the protocol.

The RCT was conducted at 19 reproductive medical centers throughout China between April 2014 and June 2017. The inclusion criteria were: (i) women with infertility aged ≤ 43 years, (ii) body mass index (BMI) under 30, (iii) had intact bilateral ovaries, and (iv) planned to undergo IVF or intracytoplasmic sperm injection (ICSI). The exclusion criteria were: (i) a history of repeated implantation failure (≥ 3 IVF or ICSI-ET cycles); (ii) any disease not suitable for ART or pregnancy (described previously) (17).

Finally, a total of 2265 eligible subjects were enrolled and randomly assigned (1:1) to receive ZYP (n=1131) or placebo (n=1134) at a dose of 5 g orally three times a day. Patients were asked to take medicine from the day of downregulation (long protocol) or from day 19-23 in a previous cycle (antagonist protocol), until the day of the pregnancy test (i.e., 2 weeks after ET). Follow-ups were carried out on the ovulation induction day, embryo transfer day, 2 weeks after transfer, 5 weeks after transfer. A telephone follow-up was performed after delivery. Treatment outcomes including rates of live birth, implantation, biochemical pregnancy, clinical pregnancy, pregnancy loss, cycle cancellation, as well as incidences of maternal, fetal, neonatal complications and neonatal weights were assessed.

### *Post hoc* analysis

Subgroups analysis was performed on the basis of clinical pregnancy rate. Odd ratios (OR) were presented to assess the efficacy. Adjusted OR were calculated using logistic regression. Subgroup analysis was performed according to baseline characteristics and cycle response details, including age, BMI, infertility type, cause of infertility, live birth history, artificial abortion history, miscarriage history, IVF history, ovulation-inducing protocol and endometrial thickness. We set 35 years old as the cutoff value for women of advanced age (32). Normal BMI was between 18.5 and 24, while BMI > 24 was defined as overweight/obesity, and BMI < 18.5 was defined as underweight (36). For endometrial thickness, 7 mm was recommended as the cutoff value based on the existing researches (37–39). Besides, Patients who had their embryos transferred were grouped according to the quality and quantity of their embryos. The efficacy outcomes evaluated in this study were rates of clinical pregnancy, implantation, biochemical pregnancy, live birth, pregnancy loss, and neonatal weights. Detailed definitions of these efficacy outcomes have been published previously (17).

Intention-to-treat (ITT) and per-protocol (PP) strategies were employed during data analyzes. For PP analysis, patients were excluded for the following reasons, drop-out or deviation to the study protocol (188 in ZYP group, 193 in placebo group), cancellation of fresh ET (314 in ZYP group, 342 in placebo group).

Categorical variables were presented as counts and proportions, and compared with χ^2^ test or Fisher’s exact test as appropriate. Odd ratio (OR) was calculated using a logistic regression model, with 95% confidence intervals (CI) presented. Age and site were selected for adjustment of OR. Neonatal birth weights were summarized as median with interquartile ranges, and analyzed using the Mann-Whitney U test. All statistical analyses were performed using the SPSS version 22.0, and P-values < 0.05 was regarded as statistically significant. Forest plots were drawn using GraphPad Prism version 9.0.

## Results

### Subgroup analysis based on rates of clinical pregnancy

**Figure 1:**
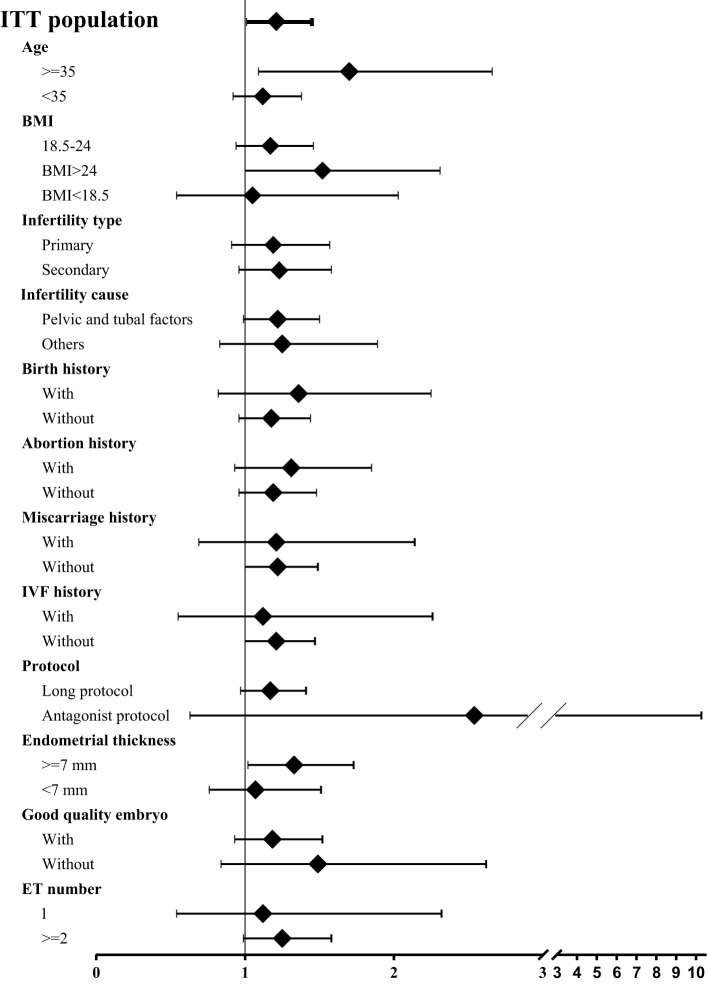
Forest plot of clinical pregnancy rates among subgroups. Odd ratio (OR) was calculated using logistic regression model, with 95% confidence intervals (CI) presented. Age and site were selected for adjustment of OR. Since not all participants underwent embryo transfer, regression and comparison among the last two subgroups (i.e., good quality embryo transfer, embryo transfer number) were calculated among population undergoing embryo transfer. A total pf 1313 participants underwent embryo transfer, 670 in ZYP group and 643 in placebo.Pregnancy outcomes in AMA subgroup.

Totally 2265 infertile women undergoing fresh embryo transfer cycles entered into the ITT analysis. In the entire population, ZYP showed a significantly higher clinical pregnancy rates than placebo (31.2% vs 27.3%, respectively; OR 1.21; 95% CI 1.01-1.45) (**Figure 1**). To test whether this effect was significant in specific subpopulations, we did a comprehensive analysis for subgroups based on the baseline characteristics. We found that ZYP was associated with significantly higher rates of clinical pregnancy than placebo among patients older than 35 years (33.0% vs 23.1%; OR 1.70; 95% CI 1.09-2.66), or patients with BMI over 24 (34.5% vs 25.0%; OR 1.52; 95% CI 1.00-2.31) (**Figure 1**). Besides, analyses in the PP population showed consistent results (**Supplementary Table 1**). Two subgroups, namely advanced maternal age subgroup (AMA) and obese/overweight subgroup, were further analyzed.

Baseline characteristic analyses showed that two treatment arms were well-balanced in AMA subgroup (**Table 1**). For AMA patients, ITT analysis demonstrated that treating with ZYP could significantly increase the rates of implantation (30.4% vs 22.4%; RR 1.36; 95% CI 1.03-1.79), biochemical pregnancy (39.8% vs 28.5%; RR 1.40; 95% CI 1.07-1.83) and clinical pregnancy (33.0% vs 23.1%; RR 1.43; 95% CI 1.05-1.95) when compared with placebo (**Table 2**). The same trend was observed in PP analysis (**Supplementary Table 2**). With regards to live birth rates, an elevated trend was observed as 26.2% vs. 19.9% [RR 1.32 (0.93-1.87)] compared with the placebo arm, without statistical significance (P=0.122).

**Table 1:** Characteristics of patients older than 35 years for the intention-to-treat analyses.

Characteristics	ZYP group (n=206)	Placebo group (n=221)	P value
Age (yr)	36.9 ± 1.9	37.0 ± 1.9	0.769
Type of infertility			
Primary	63 (30.6)	61 (27.6)	0.498
Secondary	143 (69.4)	160 (72.4)	
Duration of attempt to conceive (yr)	5.0 (3.0-8.0)	5.0 (2.0-8.0)	0.539
Concomitant infertility factors			
Pelvic factors and tubal factors	164 (79.6)	177 (80.1)	0.902
Endometriosis	10 (4.9)	2 (0.9)	0.014
Male factors	70 (34.0)	75 (33.9)	0.992
Unexplained factors	12 (5.8)	17 (7.7)	0.444
Ovulation factors	2 (1.0)	4 (1.8)	0.686
Other factors	12 (5.8)	18 (8.1)	0.349
Previous IVF cycles			
0	187 (90.8)	196 (88.7)	0.396
1	9 (4.4)	14 (6.3)	
2	6 (2.9)	4 (1.8)	
≥ 3	1 (0.5)	4 (1.8)	
Previous miscarriage	31 (15.0)	34 (15.4)	0.923
BMI (kg/m^2^)	22.1 ± 2.6	22.8 ± 3.4	0.008
AFC (n)	12 (9–16)	12 (10–15)	0.532
Serum sex hormone levels			
E_2_ (pmol/L)	140.0 (102.8-200.4)	139.5 (106.0-196.4)	0.915
FSH (international units/L)	7.2 (6.2-8.4)	7.1 (5.9-8.3)	0.389
LH (international units/L)	4.4 (3.3-5.8)	4.2 (3.1-5.5)	0.236

Data are mean ± SD, n (%) or median (interquartile range).

ZYP, Zishen Yutai Pill; yr, year; IVF, in vitro fertilization; BMI, body mass index; AFC, antral follicular count; E_2_, estradiol; FSH, follicular-stimulating hormone; LH, luteinizing hormone.

**Table 2:** Cycle responses and pregnancy outcomes of patients older than 35 years in study population for intention-to-treat analyses.

Outcomes	ZYP group (n=206)	Placebo group (n=221)	Rate ratio in ZYP group (95% CI)	P
Oocytes retrieved, no.	10 (7–14)	10 (6–14)		0.804
Matured oocytes retrieved, no.	9 (5-12)	8 (6-12)		0.550
Cleavage embryos, no.	8 (5-11)	7 (4-11)		0.359
Fertilization embryos, no.	8 (5-12)	7 (4-12)		0.735
2PN fertilization embryos, no.	7 (4-10)	6 (3-9)		0.146
Available embryo, no.	5 (3-7)	4 (2-6)		0.124
Good-quality embryos, no.	3 (0-5)	3 (1-5)		0.329
E_2_ on hCG trigger day (pmol/L)	10512.4 (6730.8-16511.3)	10437.5 (6899.6-15768.2)		0.835
Progesterone on hCG trigger day (mg/L)	0.9 (0.7–1.1)	0.9 (0.7–1.1)		0.699
Implantation rate, no./total no. (%)^#^	89/293 (30.4%)	65/290 (22.4%)	1.36 (1.03-1.79)	0.029
Biochemical pregnancy	82/206 (39.8%)	63/221 (28.5%)	1.40 (1.07-1.83)	0.014
Clinical pregnancy	68/206 (33.0%)	51/221 (23.1%)	1.43 (1.05-1.95)	0.022
Live birth	54 (26.2%)	44 (19.9%)	1.32 (0.93-1.87)	0.122
Singleton	41 (19.9%)	37 (16.7%)	1.19 (0.80-1.78)	0.398
Twin	13 (6.3%)	7 (3.2%)	1.99 (0.81-4.90)	0.125
Pregnancy loss, no./total no. (%)				
Among biochemical pregnancy	21/82 (25.6%)	15/63 (23.8%)	1.08 (0.60-1.91)	0.804
Among clinical pregnancy^†^	14/68 (20.6%)	7/51 (13.7%)	1.50 (0.65-3.45)	0.331
First trimester	9/68 (13.2%)	4/51 (7.8%)	1.69 (0.55-5.17)	0.351
Second trimester	3/68 (4.4%)	3/51 (5.9%)	0.75 (0.16-3.56)	1.000
Birth weight, g				
Singleton	3400 (3100-3600)	3250 (2900-3638)		0.263
Twin	2375 (1908-2775)	2188 (1800-2338)		0.062

Data are n (%) or median (IQR range).

ITT, intention-to-treat set; ZYP, Zishen Yutai Pill; CI, confidence interval; IQR, interquartile. P-values <0.05 were considered statistically significant.

Implantation rate = Number of gestational sacs/number of embryos transferred.

^†^In ZYP group, two case of pregnancy loss occurred, but when pregnancy loss occurred was unknown.

Part of the pregnancy outcome data has been published in the **Supplementary Materials** in our previous RCT (17).

### Pregnancy outcomes in obese/overweight subgroup

Baseline characteristic analyses showed that two treatment arms were well-balanced in this subgroup (**Table 3**). For patients with BMI > 24, significant differences were observed in the ITT population, as ZYP treatment had a higher rate of implantation (37.4% vs 28.9%; RR 1.29; 95% CI 1.02-1.64), biochemical pregnancy (39.5% vs 29.8%; RR 1.32; 95% CI 1.03-1.70), clinical pregnancy (34.5% vs 25.0%; RR 1.38; 95% CI 1.04-1.83) and live birth (29.1% vs 20.6%; RR 1.41; 95% CI 1.03-1.95) (**Table 4**). While in the PP analyses, ZYP treatment still had a better but not statistically significant pregnancy outcomes compared with placebo, which might be explained by a drastically reduced sample size in PP population (**Supplementary Table 3**).

**Table 3:** Characteristics of patients with BMI over 24 for the intention-to-treat analyses.

Characteristics	ZYP group (n=220)	Placebo group (n=248)	P value
Age (yr)	30.9 ± 4.1	31.3 ± 4.5	0.317
Type of infertility			
Primary	96 (43.6)	110 (44.4)	0.876
Secondary	124 (56.4)	138 (55.6)	
Duration of attempt to conceive (yr)	3.5 (2.0-6.0)	4.0 (2.0-6.0)	0.968
Concomitant infertility factors			
Pelvic factors and tubal factors	168 (76.4)	187 (75.4)	0.809
Endometriosis	9 (4.1)	5 (2.0)	0.188
Male factors	58 (26.4)	69 (27.8)	0.723
Unexplained factors	8 (3.6)	10 (4.0)	0.824
Ovulation factors	18 (8.2)	25 (10.1)	0.478
Other factors	22 (10.0)	20 (8.1)	0.465
Previous IVF cycles			
0	196 (89.1)	223 (89.9)	
1	11 (5.0)	16 (6.5)	0.590
2	7 (3.2)	5 (2.0)	
≥ 3	0 (0.0)	1 (0.4)	
Previous miscarriage	34 (15.5)	32 (12.9)	0.429
BMI (kg/m^2^)	26.3 ± 2.0	26.2 ± 2.2	0.534
AFC (n)	15 (12–20)	15 (11–22)	0.764
Serum sex hormone levels			
E_2_ (pmol/L)	128.5 (98.9-167.3)	126.8 (100.7-170.4)	0.959
FSH (international units/L)	6.2 (5.4-7.3)	6.3 (5.4-7.3)	0.951
LH (international units/L)	4.3 (3.0-5.8)	3.8 (2.9-5.8)	0.383

Data are mean ± SD, n (%) or median (interquartile range).

ZYP, Zishen Yutai Pill; yr, year; IVF, in vitro fertilization; BMI, body mass index; AFC, antral follicular count; E_2_, estradiol; FSH, follicular-stimulating hormone; LH, luteinizing hormone.

**Table 4:** Cycle responses and pregnancy outcomes of patients with BMI >24 in study population for intention-to-treat analyses.

Outcomes	ZYP group (n=220)	Placebo group (n=248)	Rate ratio in ZYP group (95% CI)	P
Oocytes retrieved, no.	12 (8-16)	12 (8-17)		0.806
Matured oocytes retrieved, no.	11 (8-15)	11 (8-15)		0.763
Cleavage embryos, no.	9 (6-12)	8 (5-13)		0.518
Fertilization embryos, no.	8 (5-12)	7 (4-12)		0.604
2PN fertilization embryos, no.	9 (6-13)	9 (6-13)		0.479
Available embryo, no.	4 (3-7)	5 (2-7)		0.373
Good-quality embryos, no.	4 (2-7)	3 (1-6)		0.223
E_2_ on hCG trigger day (pmol/L)	11861.4 (8224.5-16144.3)	10780.0 (7229.9-17814.2)		0.794
Progesterone on hCG trigger day (mg/L)	0.9 (0.6–1.2)	0.9 (0.7–1.2)		0.461
Implantation rate, no./total no. (%)^#^	104/278 (37.4%)	79/273 (28.9%)	1.29 (1.02-1.64)	0.035
Biochemical pregnancy	87/220 (39.5%)	74/248 (29.8%)	1.32 (1.03-1.70)	0.027
Clinical pregnancy	76/220 (34.5%)	62/248 (25.0%)	1.38 (1.04-1.83)	0.024
Live birth	64 (29.1%)	51 (20.6%)	1.41 (1.03-1.95)	0.032
Singleton	41 (18.6%)	41 (16.5%)	1.13 (0.76-1.67)	0.550
Twin	23 (10.5%)	10 (4.0%)	2.59 (1.26-5.32)	0.007
Pregnancy loss, no./total no. (%)				
Among biochemical pregnancy	18/87 (20.7%)	15/74 (20.3%)	1.02 (0.55-1.88)	0.948
Among clinical pregnancy^†^	12/76 (15.8%)	11/62 (17.7%)	0.89 (0.42-1.88)	0.760
First trimester	5/76 (6.6%)	8/62 (12.9%)	0.51 (0.18-1.48)	0.206
Second trimester	4/76 (5.3%)	3/62 (4.8%)	1.09 (0.25-4.67)	1.000
Birth weight, g				
Singleton	3535 (3163-3750)	3500 (3100-3650)		0.328
Twin	2600 (2288-2900)	2500 (2250-2800)		0.464

Data are n (%) or median (IQR range).

ITT, intention-to-treat set; ZYP, Zishen Yutai Pill; CI, confidence interval; IQR, interquartile. P-values <0.05 were considered statistically significant.

Implantation rate = Number of gestational sacs/number of embryos transferred.

^†^In ZYP group, three case of pregnancy loss occurred, but when pregnancy loss occurred was unknown.

## Discussion

In this study, subgroup analyses implied that patients at advanced maternal age (35-43 years old) or obese/overweight women (BMI 24-30) may benefit from ZYP treatment, as their rates of implantation and pregnancy outcomes were significantly increased in the ZYP group.

AMA patients are more likely to seek the help of ART due to declined fecundity, while treatment outcomes are considered inferior to those of non-advanced age patients (24). Indeed, we here found that the AMA patients had an elevated level of FSH/LH and decreased number of oocytes and quality when compared with the non-AMA population (**Supplementary Table 4**). FSH/LH ratio is recommended as one of the predictors of diminished ovarian reserve (DOR), which is defined as decreased number or quality of oocytes and the resulting subfertility (35, 40). AMA patients with DOR usually undergo a poor ovarian response to COH and are associated with unfavorable pregnancy outcomes, making it one of the most challenging tasks in IVF clinical practice (41). In this aspect, a couple of relevant recommendations toward ART for women with advanced maternal age have been made in several guidelines as well as consensus (30, 34, 35). In 2019, China has developed its first clinical practice guideline on ART strategies specifically for AMA women (35). However, ways to improve the pregnancy outcomes of this population are still limited, as evidence for several treatment recommendations is insufficient. Here, we found that AMA patients treated with ZYP had a significantly higher rates of implantation, biochemical pregnancy and clinical pregnancy than placebo, which may provide new ideas for ART strategies in AMA patients. However, no significance difference was observed with regards to live birth rates (P = 0.122). The insignificance result might be explained by a drastically reduced sample size. Notably, although without statistical significance, ZYP also exhibited beneficial effects in number of 2PN fertilization embryos (P = 0.146) and available embryos (P = 0.124). This was further supported by a recent study that ZYP exhibited beneficial effects in DOR patients undergoing IVF-ET, as the oocytes retrieved, high-quality embryos were all significantly increased in ZYP-treated patients (42). Further study is warranted to figure out the underlying mechanisms. To further overcome the above-mentioned shortcomings, a clinical trial with larger-scale has been carried out to examine the pregnancy outcomes in AMA women (Clinical Trial No. NCT03703700).

As the obesity epidemic is on the rise, the resulting decline in female fecundity poses another social problem (43). Although the effect of overweight/obesity on IVF outcomes remains inconclusive, pre-pregnancy weight loss for overweight/obese infertile women is recommended according to the first Expert Consensus on the Weight Management of Overweight/Obese Infertility Patients in China (44). Mechanistically, subfecundity caused by obesity is recognized as a collection of disorders, characterized by alterations in reproductive endocrinology, diminished ovarian reserve, reduced quality and quantity of oocyte and embryos, and endometrial receptivity (45–48). Consistent with the adverse effects of obesity on ovarian hormones discussed in the literature, overweight/obese patients included in this study showed a significantly decreased level of serum E_2_ and reduced number of oocytes retrieved (**Supplementary Table 5**). However, no significant differences were observed in E_2_ and progesterone on hCG trigger day between ZYP and placebo group, suggesting that ZYP may not exert therapeutic effects via attenuation of hormone levels. More evidences on ZYP’s effects on sexual hormone regulation is still required. Previous studies also suggested a positive role of ZYP on ovarian reserve and endometrial receptivity (18–20). These effects might account for the improved implantation and pregnancy outcomes observed in subgroup analysis regarding overweight/obese patients. Although pre-pregnancy weight loss is the most commonly used intervention for infertile overweight/obese women, damages to female fecundity may not be completely reversed. Thus, ZYP may provide a complementary treatment option for ART strategies concerning overweight and obese patients. Future studies will be carried out to confirm these findings.

There are various strengths in the current study. At first, the original cohort was a randomized, double blinded, placebo controlled and multicenter design. A long follow-up period enabled the collection of live birth outcomes and neonatal information. Due to the large sample size and long follow-up period of the RCT, the current subpopulation analysis provided a broad range of information. It should also be noted that after stratification, the baseline characteristics are still well balanced, making the two arms comparable (**Tables 1**, **3**). In addition, we conducted comprehensive statistical analysis herein, including per-protocol analysis (in **Supplementary Materials**) and logistic regression model (adjusting age and site, **Figure 1**) as sensitivity analysis. The current study provided us with valuable information for our next stage research.

The main limitation is that the current research is a *post hoc*, exploratory, and not pre-defined study setting. Although the main baseline characteristics were similar among both arms, potential confounders may also inflate the true rate difference between two groups. We did not take BMI and age into consideration in our randomization process, making the subgroup analysis less reliable. Moreover, the current analysis was underpowered to test the rate difference between two arms. In ITT analysis, we had only a power of 0.63 to test the between-group difference of clinical pregnancy rate in AMA subgroup, and a power of 0.61 in obese/overweight subgroup. Thus, the current data should be interpreted with caution.

## Conclusion

In conclusion, the current *post hoc* subgroup analysis suggested that AMA and overweight/obese women could experience clinical benefits when treated with ZYP in their fresh ET cycles. The study provides references for the use of ZYP in ART practices. However, further studies in specific subgroups should be examined in more rigorous clinical trial settings.

## Data availability statement

The raw data supporting the conclusions of this article will be made available by the authors, without undue reservation.

## Ethics statement

The studies involving humans were approved by Ethics Committee at Sun Yat-Sen Memorial Hospital. The studies were conducted in accordance with the local legislation and institutional requirements. The participants provided their written informed consent to participate in this study.

## Author contributions

XC was in charge of the clinical research and drafted the manuscript. YL participated in data analysis. NN, QH, XP participated in the clinical trial. JZ and XW participated in data analysis and manuscript revision. DY supervised the whole experiment. All authors contributed to the article and approved the submitted version.
