# Supplementary material for: Effects of the Zishen Yutai Pill compared with placebo on pregnancy outcomes among women in a fresh embryo transfer cycle: a Post Hoc subgroup analysis of a randomized controlled trial
Source: Front Endocrinol (Lausanne). 2023 Nov 21;14:1196636. doi: 10.3389/fendo.2023.1196636 (PMC10703301; doi:10.3389/fendo.2023.1196636)
Supplement: Supplementary file 1 [file DataSheet_1.pdf]

Protocol

Effect of Traditional Chinese Medicine vs. placebo on live births among women undergoing in vitro fertilization, a multicenter randomized controlled trial

[redacted]

Note: The Steering Committee and the Data and Safety Monitoring Board have discussed and revised this protocol thoroughly, including two major revisions. The final version was written on November 24, 2016, and was approved by [redacted] on March 7, 2017. After its IRB approval, this protocol has been implemented and adhered to without amendment. The Statistical Analysis Plan is contained within the protocol.

CONTENTS

1. Committee composition 5
1.1 Protocol Committee 5
1.2 Steering Committee 5
1.3 Data Coordination Committee 10
1.4 Publication Committee 10
2. Background 11
3. Objectives 12
4. Study procedures and visits 12
4.1 Study procedure flowchart 12
4.2 Screening visit 13
4.3 Start-up day visit 14
4.4 ET visit 15
4.5 Biochemical pregnancy test visit 15
4.6 Clinical pregnancy test visit 16
4.7 Delivery visit 16
5. Study design 17
5.1 General design 17
5.2 Randomization and blinding 17
5.3 Intervention 18
6. Inclusion criteria 21
7. Exclusion criteria 22
8. Physical examination 22
9. Transvaginal ultrasound scan 22
10. Laboratory tests 23
11. Outcome measures 23
12. Timeline and recruitment plan 23
13. Statistical plan 24
14. Adverse event reporting 25
14.1 Risks and discomforts 25
14.2 Adverse event definitions 28

14.3 Recording of adverse events ... 29

14.4 Causality and severity assessment ... 30

14.5 Reporting of serious adverse events and unanticipated problems ... 31

15. Concomitant medication ... 31

16. Monitoring ... 32

16.1 Data and safety monitoring ... 32

16.2 Ethics ... 32

17. Data handling and record-keeping ... 33

17.1 Data entry and case report form (CRF) ... 33

17.2 Data security ... 33

17.3 Data quality control ... 34

17.4 Audit ... 34

18. Publication policy ... 35

19. Acknowledgment section ... 36

20. Protocol revision history ... 36

21. References ... 37

1. Committee composition

1.1 Protocol Committee

Table 1. Protocol Committee

Name	Affiliation	Email
[redacted]	[redacted]	[redacted]
[redacted]	[redacted]	[redacted]
[redacted]	[redacted]	[redacted]
[redacted]	[redacted]	[redacted]
[redacted]	[redacted]	[redacted]

1.2 Steering Committee

Table 2. Steering Committee

Name	Affiliation	Email
Chair		
[redacted]	[redacted]	[redacted]

	[redacted]	
Co-investigators		
[redacted]	[redacted]	[redacted]
[redacted]	[redacted]	[redacted]
Site-investigators from 19 Hospitals		
[redacted]	[redacted]	[redacted]
[redacted]	[redacted]	[redacted]
[redacted]	[redacted]	[redacted]

	[redacted]	
[redacted]	[redacted]	[redacted]
[redacted]	[redacted]	[redacted]
[redacted]	[redacted]	[redacted]
[redacted]	[redacted]	[redacted]
[redacted]	[redacted]	[redacted]

	[redacted]	
[redacted]	[redacted]	[redacted]
[redacted]	[redacted]	[redacted]
[redacted]	[redacted]	[redacted]
[redacted]	[redacted]	[redacted]
[redacted]	[redacted]	[redacted]

	[redacted]	
[redacted]	[redacted]	[redacted]
[redacted]	[redacted]	[redacted]
[redacted]	[redacted]	[redacted]
[redacted]	[redacted]	[redacted]
[redacted]	[redacted]	[redacted]

[redacted]	[redacted]	[redacted]
[redacted]	[redacted]	[redacted]

1.3 Data Coordination Committee

[redacted] will lead the Data Coordination Committee consisting of the personnel from [redacted], including registering the trial at the Chinese Clinical Trial Registry (http://www.chictr.org/cn/). The daily operation of the Data Coordination Center is fully executed by the personnel at [redacted].

1.4 Publication Committee

[redacted]

2. Background

In vitro fertilization (IVF) is widely performed as infertility treatment and has resulted in the birth of more than 5 million infants worldwide [1]. Although significant improvements have been achieved in assisted reproductive technology (ART), clinical pregnancy rates remain variable per embryo transfer (ET). Complementary therapies, such as Traditional Chinese Medicine (TCM), are frequently used by women undergoing IVF to improve pregnancy outcomes [2-5].

Data from a prospective cohort study in the United States show that 17% of couples use TCM therapies for infertility [6]. Another cross-sectional study found that 46% of Irish patients undergoing IVF admitted regularly using TCM, with 38% having taken TCM in the 3 months before their attendance for treatment [7].

Previous studies suggested a potential benefit from TCM in improving IVF outcomes [8-9]. A meta-analysis of 40 trials found a 2-fold increase in the clinical pregnancy rate in TCM users compared with controls [10]. On the other hand, there were significant methodological limitations in the studies included in that meta-analysis, which affected the evidence's quality. For example, the study sample sizes were small, there were few randomized controls, and studies lacked the key outcome, i.e., live birth. These limitations have hampered the development of recommendations for clinical practice and highlighted the need for a well-designed randomized controlled trial (RCT) to address the effect of TCM in ART [11-13].

3. Objectives

The objective of this multicenter RCT is to assess the efficacy and safety of TCM vs. placebo during IVF or intracytoplasmic sperm injection (ICSI) on the pregnancy outcomes.

4. Study procedures and visits

4.1 Study procedure flowchart

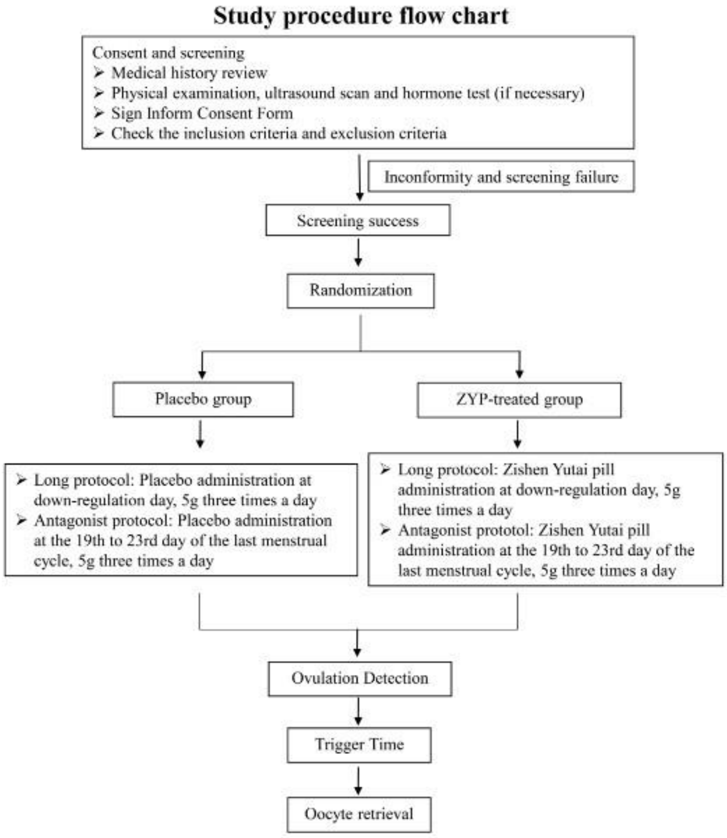

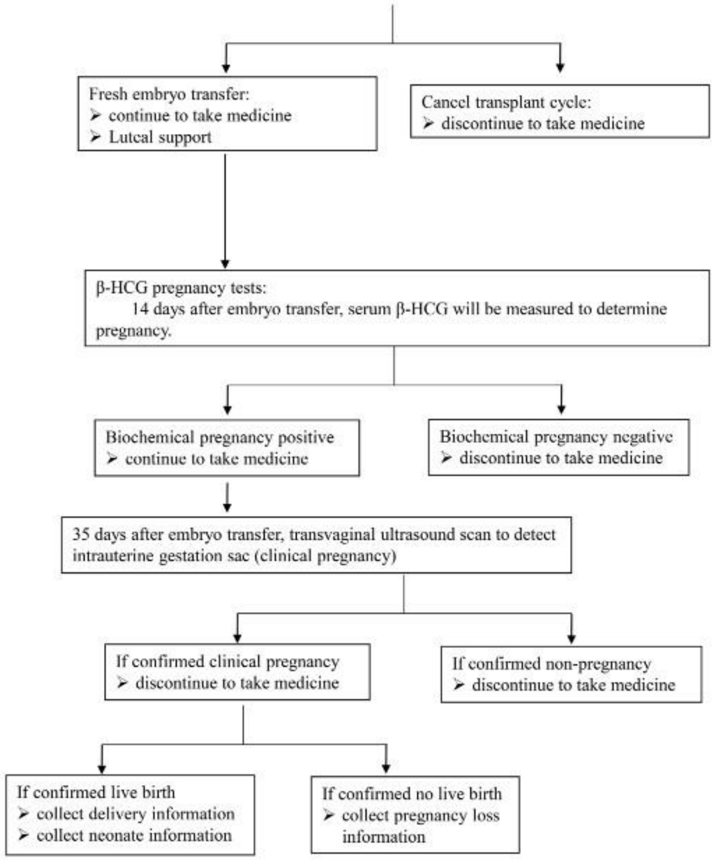

4.2 Screening visit

After completing all IVF/ICSI preparation tests and organizing the medical record of ART, the screening visit will be performed. Assisted reproductive technology physicians will refer the patients to the investigators. During the screening visit, the following procedures will be completed.

(1) Obtaining the signed informed consent form.

(2) Collecting baseline information, including age, medical record number, and birth date.

(3) Reviewing the medical history, including infertility history, childbearing history, gynecological surgery history, history of past illness, and menstrual history.

(4) Carrying out a physical examination including height, weight, abdominal circumference, hip circumference, medical examination, and gynecologic examination.

(5) Performing a TCM dialectical evaluation for premedication assessment.

(6) Measuring the baseline sex hormones, including estradiol (E_2), follicle-stimulating hormone (FSH), luteinizing hormone (LH), prolactin (PRL), and testosterone (T).

(7) Reviewing the previous ET/FET cycle record;

(8) Going through all the safety test results for assisted reproductive technique and pregnancy to determine if any exclusion criteria are met. For example, repeated implantation failure, severe endometriosis, untreated bilateral fallopian tube hydrops, unhealed endometrium diseases, and diseases unsuitable to carry out assisted reproductive technology or conceive at present.

(9) Dispensing condition.

4.3 Start-up day visit

The start-up day visit will take place when the participants have a good health condition for oocyte retrieval. During the start-up day visit, the following procedures will be completed.

(1) Qualification confirmation.

(2) Adverse events and concomitant medication record by filling in the adverse event record form and concomitant medication record form.

(3) Medication on start-up day, including trade name, dose, duration, and total amount.

(4) Basal serum hormone levels, including E_2, FSH, LH, and P (progesterone).

(5) Dispensing condition.

4.4 ET visit

The ET and medication information will be recorded using a form designed for this visit. During the ET visit, the following procedures will be completed.

(1) Qualification confirmation.

(2) Adverse events and concomitant medication record by filling in the adverse event record form and concomitant medication record form.

(3) Summary of ovulation induction medication including Gn, HMG, LH, and others.

(4) B-mode ultrasound and basal serum hormonal level.

(5) Information about oocytes retrieval and transferred embryos.

(6) Information about the medicines used in luteal phase support, including trade name, dose, duration, and total amount.

(7) Dispensing condition.

4.5 Biochemical pregnancy test visit

At 14 days after transplantation, a biochemical pregnancy test will be performed. Adverse events and concomitant medication will be recorded by filling in the adverse event record form and concomitant medication record form. If serum β-HCG levels are $\geq$10 IU, biochemical pregnancy will be called, and the form will be filled in. At last, the dispensing

condition will be summarized.

4.6 Clinical pregnancy test visit

At 5 weeks after transplantation, B-mode ultrasound will be performed, and a clinical pregnancy test will take place. The presence of an intrauterine gestational sac will confirm the clinical pregnancy. Adverse events and concomitant medication will be recorded by filling in the adverse event record form and concomitant medication record form. In addition, drug recovery will be summarized. TCM dialectical evaluation for post-medication assessment will be performed. If a participant does not have a clinical pregnancy, then the TCM dialectical evaluation will be completed at the biochemical pregnancy visit. If a clinical pregnancy is confirmed, the investigator must check contact information with participants. Furthermore, the investigator needs to ask the participants to notify when and where to go to the hospital for delivery.

4.7 Delivery visit

When the participant is preparing to deliver, the investigator will collect the delivery information and infant information according to the form designed for this visit. The delivery information mainly includes the delivery mode and pregnancy complications. The infant information mainly includes sex, weight, length, and birth defect. If the participant delivers in the center, the obstetric medical record will be checked. If the participant delivers at another hospital, a telephone follow-up will be performed.

Whether the participant delivers or has an abortion, the final pregnancy outcome record form

designed for this visit will need to be completed.

5. Study design

5.1 General design

It will be a multicenter, prospective, randomized (1:1 treatment ratio) clinical trial comparing the live birth rate of a fresh ET in 2265 infertile patients undergoing their present cycle of IVF or ICSI. COH-qualified patients will be randomized into either of two groups: Group A and Group B. The COH protocol will be selected for the patients according to clinical routine. All the participants will receive a standardized GnRH agonist long protocol or GnRH antagonist protocol and standardized luteal phase support.

5.2 Randomization and blinding

The participants will be randomized 1:1 to receive double-blind single-dummy monotherapy with placebo or ZYP, using a specific computer system. The randomization will be stratified by the study site. Patients, investigators and clinical staff performing this trial will be blinded to treatment allocation. The package and contour are the same in the placebo and TCM pills. Blinding will be maintained until the completion of the analysis. Unblinding would be allowed in case of a medical emergency. The cause and time of unblinding would be recorded in detail and signed by the treating physicians.

When all live birth information is retrieved, the computer system will disclose the placebo group or TCM group.

5.3 Intervention

5.3.1 Zishen Yutai Pill

The TCM used in this study is called the Zishen Yutai Pill. It contains 15 Chinese traditional medicinal herbs. As a listed TCM formula, the Zishen Yutai Pill is produced with specified prescription and technology. The dosage of the medicinal material with the corresponding preparation amount is fixed (Table 3). In addition, the production process of the Zishen Yutai Pill complies with the relevant requirements of China's drug administration law and GMP. The production process is normative and controllable to ensure the quality consistency of each batch of products.

Table 3. Zishen Yutai Pill standard prescription and raw material amount in a daily dose

Medicine material	Standard prescription amount/g	Raw material amount in daily dose/g
Cuscutae Semen	800	9.604
Ginseng radix et rhizoma	50	0.600
Dipsaci radix	480	5.762
Taxilli herba	480	5.762
Eucommiae cortex	290	3.481

Morindae officinalis radix	190	2.281
Cervi cornu degelatinatum	140	1.681
Codonopsis radix	580	6.963
Atractylodis macrocephalae rhizome	240	2.881
Asini corii colla	30	0.360
Lycii fructus	190	2.281
Rehmanniae radix praeparata	480	5.762
Polygoni multiflori radix praeparata	240	2.881
Artemisiae argyi folium	140	1.681
Amomi fructus	70	0.840

5.3.2 Composition and manufacturing process of the placebo

1) Composition: Pregelatinized starch, microcrystalline cellulose, black iron oxide, refined honey, dextrin.

2) Manufacturing process: Pregelatinized starch, microcrystalline cellulose, and black iron oxide are uniformly mixed, crushed, and sieved, and then the refined honey is added to the mixed powder to make the wet pill with the required size. After drying, it is coated with a mixture of black iron oxide and talc powder, 3% dextrin solution, 75% ethanol solution, and

refined honey. Finally, eligible pills are polished using Chinese insect wax, selected, and packaged.

5.3.3 Ovulation induction protocol

(1) GnRHa long protocol: triptorelin (Decapeptyl; Ferring, Switzerland) will be started on day 21 of the preceding menstrual cycle, subcutaneously. After 14 days of downregulation, the follicle-stimulating hormone will be started, which will continue until the day of human chorionic gonadotropin (hCG) administration. The study medicine will be introduced on the day of downregulation, three times per day, with a dose of 5 g until the day of the pregnancy test 2 weeks after ET.

(2) GnRH antagonist protocol: Follicle-stimulating hormone will be administered daily beginning on day 2 of the cycle. The GnRH antagonist (Cetrotide, Serono, Geneva, Switzerland) will be given at a daily dose of 0.25 mg subcutaneously when the leading follicle diameter measures 12-14 mm. Study medicine cotreatment will be introduced on day 20 in a previous cycle three times per day at a dose of 5 g until the day of the pregnancy test 2 weeks after ET.

When the leading follicle diameter reaches 18 mm or greater, ovulation will be triggered with 10,000 IU of hCG intramuscularly. Serum progesterone, LH, and E_2 will be analyzed on the day of hCG administration.

5.3.4 Oocyte retrieval and fresh ET cycles

Oocyte retrieval will be done 35-36 h following hCG administration. Intramuscular progesterone at a daily dose of 40 mg will be given as luteal phase support on the day of oocyte retrieval. The oocytes will be inseminated approximately 4-6 h after follicular aspiration by IVF or ICSI, according to sperm quality.

All participants will undergo fresh ET. ET will be performed 3 days after oocyte retrieval. The maximum number of embryos transferred is three. Morphologic criteria will be used for embryo scoring. The high-quality embryos are defined as the cleavage stage embryo at level I. The cleavage-stage embryo at level I is defined as uniform cells in size and cytoplasm, with 0-10% cell debris and no multinucleation.

After ET for 2 weeks (14 days), biochemical pregnancy will be determined by serum β-hCG measurement. If biochemical pregnancy is confirmed, clinical pregnancy will be determined according to the ultrasound scan after ET for 5 weeks (35 days). Study intervention will be stopped if the pregnancy test is β-hCG negative (biochemical pregnant negative). For those with positive results in biochemical pregnancy test, study intervention will continue until clinical pregnancy is confirmed by ultrasound.

6. Inclusion criteria

(1) Infertile women aged ≤43 years

(2) Pro-conceptive patients who planned to perform IVF/ICSI-ET (long protocol and antagonist protocol).

(3) Body mass index ≤30 kg/m^2.

(4) With ovaries on both sides.

7. Exclusion criteria

(1) Repeated implanting failures (≥3 IVF/ICSI–ET failure cycles).

(2) Severe endometriosis, including adenomyosis of the uterus and ovarian chocolate cyst.

(3) Untreated bilateral hydrosalpinx.

(4) Untreated endometrial diseases.

(6) Disease that not suitable for assisted reproduction or not suitable for pregnancy.

8. Physical examination

A physical examination will be conducted on all participants by an investigator. The parameters include height, weight, abdominal circumference, and hip circumference, and they will be recorded to the nearest 0.1 cm, 0.1 kg, and 1 cm. Height and weight will be measured without shoes, and patients will be dressed in light clothing. Abdominal girth will be measure at the umbilicus level, and hip circumference will be at the widest diameter.

9. Transvaginal ultrasound scan

An ultrasound scan with a transvaginal probe will be carried out. Uterine dimension, endometrial thickness and type, bilateral ovarian dimension, and follicle number will be measured through the ultrasound scan. The uterine size will be determined at the widest diameter. The investigator will determine endometrial types. Endometrial thickness is the largest anterior-posterior measurement of the endometrium in the sagittal plane. Ovarian dimension is measured by analysis of the largest plane of the ovary in two dimensions. In

addition, antral follicle count (AFC) will be recorded.

10. Laboratory tests

Hormone and pregnancy tests will be performed at the local laboratories. In addition, blood work will be performed on start-up day, hCG day, and 2 weeks after ET. The blood sample (5 ml) will be collected with an anticoagulant tube for DNA analysis, metabolomics analysis, and repository, which will be stored at -80°C. In addition, 5 ml of follicular fluid will be sampled on the oocyte retrieval day. Total T (TT), LH, FSH, PRL, E_2, and progesterone will be measured with a chemiluminescence immunoassay (Beckman Coulter kit, Beckman Coulter Dxl800, Inc., USA). The intra- and inter-assay coefficients of variances for TT are 1.99% and 4.22 %; for LH, they are 3.8% and 4.6%; for FSH, they are 3.5% and 4.1%; for E2, they are 1.3% and 3.5%; for progesterone, they are 6.1% and 6.5%; and for PRL, they are 1.61% and 4.4%.

11. Outcome measures

The live birth rate is the primary outcome. The secondary outcomes include the rates of implantation, biochemical pregnancy, clinical pregnancy, pregnancy loss, cycle cancellation, and adverse events.

12. Timeline and recruitment plan

The planned duration of recruitment will be 24 months with 19 centers. The enrollment target of our clinical trials is 2265 randomized participants. The number of participants taken in

each center will be allocated according to the actual situation. The treatment period may need about 3 months and another 9 months to trace the pregnancy outcome. In short, a total of 36 months will be required to complete this trial, from initial recruitment to pregnancy outcome period.

13. Statistical plan

a) Sample size determination

We conducted a pilot study to collect preliminary data on the clinical pregnancy rate of ZYP (38.3%) versus placebo (31%) to guide our sample size estimation. Since the live birth rate is usually about 10% lower than the clinical pregnancy rate [14], we calculated that at least 960 patients per study group were required to compare the live birth rates, achieving a power above 80% at a significance level of 0.05. We increased the sample size from 960 to 1130 to allow for a dropout rate of 15%

b) Statistical Methods

We will employ the intention-to-treat (ITT) strategy for our primary data analysis. Per-protocol (PP) analysis will be performed by excluding the participants who drop out of the study, fail to comply with the study protocol, or cancel ET.

For the comparison of baseline characteristics, categorical data will be presented as frequency and percentage, and the between-group differences will be assessed using the chi-square test or Fisher's exact test for expected frequencies of <5. Continuous data will be expressed as means ± standard deviation and will be analyzed using t-test or the Wilcoxon rank-sum test.

All statistical calculations will be done using SPSS 19 (IBM, Armonk, NY, USA) for Microsoft Windows. P-values <0.05 will be considered statistically significant.

c) Interim analysis

We propose to do an interim analysis at 25% of target enrollment to avoid serious deviation from clinical habits of local PIs from our standard protocol. The final data analysis will be completed after all live births in the trial.

14. Adverse event reporting

14.1 Risks and discomforts

Compared with the usual IVF patients, participating in this study will not increase additional risks. The possible risks and discomforts in common IVF technology are detailed in the informed consent, including in vitro fertilization ET, embryo freezing, and so on. The table below lists all procedures, including related risks and discomforts.

Table 4. Risks and discomfort

**Procedures and events**	**Risks and discomfort**
Controlled ovarian hyperstimulation (COH)	Frequent subcutaneous injection, frequent venipuncture, frequent transvaginal ultrasound scan. Supra-physiologic E_2 may increase the risk of cancer ovary torsion or ovary rupture.
Ovarian hyperstimulation	Massive enlargement of the ovaries, ascites, bloating,

syndrome (OHSS)	nausea, and vomiting. Severe cases may have thoracic edema, breathing difficulties, oliguria, even anuria, and may require hospitalization, medication, or puncture drainage. A very severe case may suffer from thrombosis, damage to the liver or renal function, and even death.
Oocyte retrieval	Anesthesia accident, pelvic organ injury, intra-abdominal hemorrhage, puncture site hemorrhage, in serious case surgery or transfusion may be needed, infection.
ICSI	Microinjection may injure an oocyte, pass an unknown disease gene to the next generation.
Embryo transfer	Infection.
Embryo frozen and thaw	Embryotic development arrest. The survival rate of thawed embryos is 95%.
Standard venipuncture for blood work	Slight pain, ecchymosis at the site of puncture, infection, or bleeding at the site.
Transvaginal ultrasound	Abdominal or pelvic discomfort.
Ectopic pregnancy	May require medical or surgical treatment. In severe cases, pregnancy site rupture can result in intra-abdominal hemorrhage, even shock, or death if treatment is delayed.
Multiple pregnancies	May require embryo reduction, increase risk of pregnancy complication, fetus abnormalities, and preterm delivery.

Infertility treatment	Anxiety or emotional distress to various degrees.
Zishen Yutai Pills	Unclear side effects and contraindications. It has been reported that some patients who took the Zishen Yutai pill suffered from nausea, dry mouth, and constipation that disappeared after drug withdrawal.

The participants are not expected to have all of these complications, and they will be allocated to a treatment group at random. The treatment may be less effective or have more complications than the other research treatment.

In this study, the long protocol and GnRH antagonist protocol will be performed according to each patient's actual situation. The GnRH antagonist protocol will be used to minimize the risk of OHSS. The initial dose will be determined according to the age, basal FSH level, basal AFC, weight, and previous situation promoting ovulation. The gonadotropin (Gn) dose will be individualized according to the development of follicle and serum E_2 level. In addition, in case of high ovarian response, the cycle will be canceled to avoid OHSS. If there are three or more fetuses, then a reduction will be performed to minimize the risks of multiple pregnancies. A responsible investigator or a resident doctor on 24 h call can be contacted at each site if any adverse event occurs during this study.

Every effort will be taken to avoid injury as a result of participation. If adverse events occur, active treatment will be provided. Furthermore, if a medical dispute is involved, it will be disposed of as a routine medical event.

14.2 Adverse event definitions

The side effects and contraindications of the Zishen Yutai Pill are not clear. However, it has been reported that some patients who took the Zishen Yutai Pill suffered from nausea, dry mouth, and constipation that disappeared after drug withdrawal.

Adverse event means any untoward or unfavorable medical occurrence associated with the subject's participation in the research, whether or not considered related to the study intervention. **Adverse events can be any of the following:**

• Physical signs or symptoms, including medication side effects.

• Abnormal laboratory values.

• Changes in vital signs, physical exam findings, or test results.

• An increase in the frequency or intensity (worsening) of a condition or illness presents before study enrollment.

In this trial, adverse events will not include:

• Pre-existing conditions or illnesses that do not worsen during the study period (record these in the medical history).

• Normal conditions associated with pregnancy.

• Miscarriages which occur up to 20 weeks.

Serious adverse event: Any event temporally associated with the subject's participation in research that meets any of the following criteria:

• Death.

• Life-threatening (at immediate risk of death).

• Severely or permanently disabling.

• Requires in-patient hospitalization or prolongation of existing hospitalization.

• Pregnancy loss after 20 weeks gestation.

• Results in a congenital anomaly/birth defect.

• Or any event so deemed as serious by the PI at the site.

Note: A "severe" adverse event is not the same as a "serious adverse event" or SAE. Severity is based on the event's intensity, whereas seriousness is based upon the event outcome as it poses a threat to the patient's life or functioning.

Unexpected, adverse event: A adverse event is considered "unexpected" if it is not listed in the general investigational plan or protocol or is not listed at the specificity or severity that has been previously observed and/or specified.

14.3 Recording of adverse events

All adverse events will be observed during the clinical trial. The investigators will require the participants to reflect the change in patients' condition truthfully after using the drug and avoid suggestive questions. Adverse events and unexpected side effects (including symptoms, signs, and laboratory tests) will be observed while observing the curative effect. In order to determine whether adverse events are associated with the experimental drug, they will be recorded in the CRF in detail, including the occurrence time, symptoms, signs, degrees, duration, laboratory examination indicators, treatment methods, procedures, results, follow-up time, and so on. Combined medication will be recorded in detail to analyze the correlation

between adverse events and experimental drugs. In addition, the record will be signed and dated.

When adverse reactions occur, the investigator will take necessary measures, such as adjusting the dose, temporarily discontinuing the medication, and decide whether to terminate the trial or not. If a serious adverse event occurs, the unit undertaking the study must immediately take necessary treatment measures to protect the subject's safety.

14.4 Causality and severity assessment

According to the documented adverse events and abnormal test findings, the investigator will need to determine that if the abnormal test finding should be classified as an adverse event and if the adverse events are related to the study intervention or meet the criteria for a serious adverse event. The relationship between the experimental drug and adverse events are divided as "must be related to", "probably be related to", "maybe related to", "may not be related to", "remain be evaluated," and "cannot be evaluated". The level of adverse event response will be evaluated and reported as follows:

Mild: The participant can tolerate the event, and it does not affect the treatment. It need not take special actions and is not harmful to the participant.

Moderate: The participant is intolerant and requires withdrawal or special treatment, which has a direct impact on their health.

Severe: It is a life-threatening, fatal, or disabling event and requires withdrawal or emergency treatment immediately.

14.5 Reporting of serious adverse events and unanticipated problems

Whether or not related to the study drugs, when serious adverse events appear during the trial, the investigator should rescue timely. It should be reported to the primary investigator in the trial center timely. Moreover, it should be reported to the clinical trial leader unit ethics committee within 24 h. The investigator will have to document this serious adverse event and take necessary measures to ensure the participants' safety and interests. It should also be timely reported to the pharmaceutical supervisory and administrative department and the administrative department of health. At the same time, it should be notified to the investigator involved in the same clinical trials. If it is confirmed as a serious adverse event related to the experimental drug, the investigator will bear the rescue and treatment cost and the corresponding economic compensation.

The investigator must fill in the "serious adverse events report form". When, how and to whom the serious adverse events are reported should be recorded in the original data.

15. Concomitant medication

The following medications will be allowed during this study, and concomitant medication should be recorded.

(1) Anti-diabetic agents and anti-hypertension agents.

(2) Folic acid supplement aimed at preventing neural tubal defect.

(3) For patients with abnormal bleeding/prolonged amenorrhea, progestin, micronized progesterone, or dydrogesterone.

(4) During controlled ovarian stimulation, hMG will be allowed to use in patients with slow

E_2 increase or follicles development.

(5) For patients with moderate or severe OHSS, routine clinical treatment, such as fluid infusion, albumin infusion, aspirin, or preventive antibiotics, will be used.

(6) For patients with threatening abortion, an extra dose of progesterone will be allowed to use. Concomitant medication will be recorded.

(7) For patients with pregnancy complications, clinical standard care will be performed. Concomitant medication will be recorded.

16. Monitoring

16.1 Data and safety monitoring

The clinical trial management office will review and interpret data generated from the study and review the protocol's revisions before their implementation. Its primary objectives are to ensure the safety of study subjects and the integrity of the research data. The office will advise on research design issues, data quality and analysis, and research participant protections for the study. The office will hold regular conference calls in English to review the protocol for ethical and safety standards, monitor the trials' safety, monitor the data's integrity for original study design, and provide advice on study conduct. The office will review the trial's progress, adjudicate adverse events, and decide on any premature closure of the study. The DCC will coordinate the call and provide study updates before the call via email.

16.2 Ethics

Ethics approval has been sought from the [redacted]

[redacted] Ethics approval will be obtained from each participating center.

17. Data handling and record-keeping

[redacted]

17.1 Data entry and case report form (CRF)

The trial investigators must go through GCP training and understand the protocol and relevant information in advance adequately. The protocol will be executed strictly, and clinical trial drugs will be provided to qualified subjects after screening. All data required on the CRF must be recorded. If there is an error, please underline it with a line and refill in the correct data and the correct person's initials and date. Do not cover up error data, and do not use erasers or correction fluid to cover or draw lines. Each page of the CRF must be completed, and all items should be filled in. Fill "×" in "□" means to select this item. If this item is "not done", then enter "ND". "Unknown" is "UK". "Not available," or "not applicable" is "NA". CRFs will be implemented in a form through hand-writing.

17.2 Data security

[redacted]

[redacted], will take the responsibility to establish the project space in the CRF records, establish individual folders for each sub-center and assign the jurisdiction to the users. The database managers have the highest jurisdiction to manage and monitor the data and actions. The users of each sub-center will be allowed to enter their patients' information and study results in their folders. The database managers take the responsibility to decide which data could be disclosed to the public. Patients' private information includes names, age, and telephone numbers, will be critically protected and will never be allowed to disclose.

17.3 Data quality control

Quality control of the data will be handled at three different levels. The first level is the real-time logical and range checking built into the database. The research coordinators and data entry clerks at the participating sites must ensure data accuracy as the first defense. The second is the remote data monitoring and validation that is the primary responsibility of the data manager and programmer at the DCC. The data manager will conduct monthly comprehensive data checks, as well as regular manual checks. Manual checks will identify more complicated and less common errors. The data manager will query sites until each irregularity is resolved. The third level of quality control will be the site visits, where data in our database will be compared against source documents. Identified errors will be resolved between the DCC and clinical sites. The visits will assure data quality and patient protection.

17.4 Audit

Another security measure is the audit. It will ensure that only authorized additions, deletions,

or alterations of information in the electronic record have occurred and allows a means to reconstruct significant details about study conduct and source data collection necessary to verify data quality and integrity. Computer-generated, time-stamped audit trails will be implemented for tracking changes to electronic source documentation.

Controls will be established to ensure that the system's date and time are correct. This project is a multicenter clinical trial taking place in China. System documentation will explain time zone references as well as zone acronyms. Dates and times will include the year, month, day, hour, and minute to the date provided by international standard-setting agencies. The ability to change the date or time will be limited to authorized personnel, and such personnel will be notified if a system date or time discrepancy is detected.

In addition to the internal safeguards built into the computerized system, external safeguards will be implemented. Data will be stored at the servers housed at [redacted]. Records will be regularly backed up, and record logs are maintained to prevent a catastrophic loss and ensure the data's quality and integrity.

18. Publication policy

Table 5 Authorship order category

1	[redacted]
2, n-2	[redacted]
n-1	[redacted]
n	[redacted]

It is anticipated, there will be up to 20 authors per major manuscript. The authorship order for the participating sites will be based upon subject recruitment, data accuracy, and promptness of data report and will start at the position 2 and go to position n-2. Data accuracy will be ranked according to the rate of missing or false data entries/randomized subject at each site. Inquires that show data was accurately entered will not count against this rate of data inaccuracy. Each site's PI will be responsible for documenting the contributions to the study of that site's authors. We encourage the site investigators to establish the second hypothesis and have publications by sharing these data under the publication committee's supervision.

19. Acknowledgment section

The acknowledgment section will include other investigators and study personnel who contributed substantially to the study by site and members of the advisory board and Data Safety Monitoring Board. The designation will list the initials of the individual, followed by their highest degree. Significant contributions include but are not limited to protocol review, initiation and participation at each site, subject recruitment and enrollment, study conduct, data analysis, and manuscript preparation.

20. Protocol revision history

[redacted] After the initial version was prepared by the protocol committee, this protocol underwent two major internal revisions by the investigators. The final version was completed on November 24, 2016 and submitted for

the IRB approval.

Date written **July 5, 2013**

Version **1**

Date revised **December 8, 2014**

Version **2**

Date revised **November 24, 2016**

Version **3**

21. References

[1] Kissin DM, Jamieson DJ, Barfield WD. Monitoring health outcomes of assisted reproductive technology. N Engl J Med. 2014, 371: 91-93. doi: 10.1056/NEJMc1404371

[2] Smith JF, Eisenberg ML, Millstein SG, et al The use of complementary and alternative fertility treatment in couples seeking fertility care: data from a prospective cohort in the United States. Fertil Steril. 2010, 93(7): 2169–2174. doi: 10.1016/j.fertnstert.2010.02.054

[3] Coulson C, Jenkins J. Complementary and alternative medicine utilization in NHS and private clinic settings: a United Kingdom survey of 400 infertility patients. J Exp Clin Assist Reprod. 2005, 2(1):5. doi: 10.1186/1743-1050-2-5

[4] Rayner JA, Willis K, Burgess R. Women's use of complementary and alternative medicine for fertility enhancement: a review of the literature. J Altern Complement Med. 2011, 17(8): 685–690. doi: 10.1089/acm.2010.0435

[5] Stankiewicz M, Smith C, Alvino H, et al. The use of complementary medicine and therapies by patients attending a reproductive medicine unit in South Australia: a prospective

survey. Aust N Z J Obstet Gynaecol. 2007, 47(2):145–149. doi: 10.1111/j.1479-828X.2007.00702.x

[6] Smith JF, Eisenberg ML, Millstein SG, et al. The use of complementary and alternative fertility treatment in couples seeking fertility care: data from a prospective cohort in the United States. Fertil Steril, 2010, 93(7): 2169–2174. doi: 10.1016/j.fertnstert.2010.02.054

[7] Shannon J, El Saigh I, Tadrous R, et al. Usage of herbal medications in patients undergoing IVF treatment in an Irish infertility treatment unit. Ir J Med Sci, 2010, 179(1): 63–65. doi: 10.1007/s11845-009-0378-5

[8] Zhu WJ, Li XM, Chen XM, et al. Effect of Zishen Yutai pill on embryo implantation rate in patients undergoing fertilization embryo transfer in vitro. Chin J Integr Med. 2002, 22(10), 729-737. doi: 10.3321/j.issn:1003-5370.2002.10.002

[9] Zhou L, Zhou J, Jiang J, et al. Reproductive toxicity of Zishen Yutai pill in rats: Perinatal and postnatal development study. Regul Toxicol Pharmacol. 2016, 81: 120-127. doi: 10.1016/j.yrtph.2016.07.015

[10] Chinese herbal medicine for female infertility: An updated meta-analysis. Karin Ried, Complement Ther Med, 2015, 23(1): 116-128. doi: 10.1016/j.ctim.2014.12.004

[11] See CJ, McCulloch M, Smikle C, et al. Chinese herbal medicine and clomiphene citrate for anovulation: a meta-analysis of randomized controlled trials. J Altern Complement Med 2011, 17(5): 397-405. doi: 10.1089/acm.2010.0254

[12] Tan L, Tong Y, Sze SCW, et al. Chinese herbal medicine for infertility with anovulation: a systematic review. J Altern Complement Med. 2012, 18(12):1087-1100. doi: 10.1089/acm.2011.0371

[13] Zhang J, Li T, Zhou L, et al. Chinese herbal medicine for subfertile women with polycystic ovarian syndrome. Cochrane Database Syst Rev. 2010, 9: CD007535. doi: 10.1002/14651858.CD007535.pub2

[14] Olivius K, Friden B, Lundin K, et al. Cumulative probability of live birth after three in vitro fertilization/intracytoplasmic sperm injection cycles. Fertil Steril. 2002, 77(3): 505-510. doi:10.1016/s0015-0282(01)03217-4
