# Supplementary material for: Effects of the Zishen Yutai Pill compared with placebo on pregnancy outcomes among women in a fresh embryo transfer cycle: a Post Hoc subgroup analysis of a randomized controlled trial
Source: Front Endocrinol (Lausanne). 2023 Nov 21;14:1196636. doi: 10.3389/fendo.2023.1196636 (PMC10703301; doi:10.3389/fendo.2023.1196636)
Supplement: Supplementary file 2 [file Table_1.docx]

**Effects of the Zishen Yutai Pill Compared with Placebo on Pregnancy Outcomes among Women in a Fresh Embryo Transfer Cycle: A Post Hoc Subgroup Analysis of a Randomized Controlled Trial**

**Supplementary Table 1. Clinical pregnancy rate in different subgroups for per-protocol analyses**

| **Outcome** | **ZYP group**  **(n=625)** | **Placebo Group**  **(n=594)** | **P** | **Adjusted OR**  **(95% CI)** |
| --- | --- | --- | --- | --- |
| **PP population** | 351/625 (56.2%) | 307/594 (51.7%) | 0.117 | 1.23 (0.98, 1.55) |
| **Age** |  |  |  |  |
| >=35 | 67/127 (52.8%) | 51/128 (39.8%) | 0.039 | 1.66 (0.97, 2.82) |
| <35 | 284/498 (57.0%) | 256/466 (54.9%) | 0.513 | 1.12 (0.87, 1.46) |
| **With abnormal BMI** |  |  |  |  |
| 18.5=<BMI=<24 | 250/440 (56.8%) | 214/418 (51.2%) | 0.099 | 1.26 (0.96, 1.66) |
| BMI＞24 | 75/134 (56.0%) | 61/121 (50.4%) | 0.374 | 1.18 (0.69, 2.01) |
| BMI＜18.5 | 26/50 (52.0%) | 31/54 (57.4%) | 0.580 | 0.70 (0.27, 1.82) |
| **Infertility type** |  |  |  |  |
| Primary infertility | 150/279 (53.8%) | 139/259 (53.7%) | 0.982 | 1.04 (0.73, 1.48) |
| Secondary infertility | 201/346 (58.1%) | 168/335 (50.1%) | 0.038 | 1.43 (1.05, 1.95) |
| **Cause of infertility** |  |  |  |  |
| Pelvic and tubal factors | 281/491 (57.2%) | 250/470 (53.2%) | 0.208 | 1.20 (0.93, 1.56) |
| Others | 70/134 (52.2%) | 57/124 (46.0%) | 0.314 | 1.50 (0.89, 2.55) |
| **Live birth history** |  |  |  |  |
| With | 59/103 (57.3%) | 42/92 (45.7%) | 0.105 | 1.72 (0.91, 3.26) |
| Without | 292/522 (55.9%) | 265/502 (52.8%) | 0.312 | 1.16 (0.90, 1.49) |
| **Artificial abortion history** |  |  |  |  |
| with | 107/174 (61.5%) | 93/188 (49.5%) | 0.021 | 1.74 (1.12, 2.71) |
| without | 244/451 (54.1%) | 214/406 (52.7%) | 0.683 | 1.08 (0.82, 1.42) |
| **Miscarriage history** |  |  |  |  |
| With | 44/77 (57.1%) | 37/68 (54.4%) | 0.741 | 1.39 (0.64, 3.03) |
| Without | 307/548 (56.0%) | 270/526 (51.3%) | 0.123 | 1.25 (0.98, 1.60) |
| **IVF history** |  |  |  |  |
| With | 24/66 (36.4%) | 25/50 (41.7%) | 0.542 | 0.87 (0.38, 1.98) |
| Without | 327/558 (58.6%) | 282/534 (52.8%) | 0.054 | 1.29 (1.01, 1.65) |
| **Stimulation protocol** |  |  |  |  |
| Long protocol | 339/601 (56.4%) | 303/570 (53.2%) | 0.264 | 1.17 (0.93, 1.49) |
| Antagonist protocol | 12/24 (50.0%) | 4/24 (16.7%) | 0.014 | 2.53 (0.52, 12.46) |
| **Endometrium thickness** |  |  |  |  |
| >=7 mm | 177/320 (55.3%) | 149/297 (50.2%) | 0.201 | 1.22 (0.88, 1.69) |
| <7 mm | 102/191 (53.4%) | 93/177 (52.5%) | 0.869 | 1.11 (0.72, 1.71) |
| **Good quality embryo transfer** |  |  |  |  |
| With | 300/517 (58.0%) | 269/494 (54.4%) | 0.179 | 1.20 (0.93, 1.55) |
| Without | 51/108 (47.2%) | 38/100 (38.0%) | 0.252 | 1.43 (0.79, 2.57) |
| **ET number** |  |  |  |  |
| 1 | 28/75 (37.3%) | 23/64 (35.9%) | 0.865 | 0.93 (0.44, 1.99) |
| >=2 | 323/550 (58.7%) | 284/530 (53.6%) | 0.089 | 1.29 (1.01, 1.65) |

1. Abnormal BMI was defined as >24 or <18.5.

2. Odd ratio (OR) was calculated using logistic regression model, with 95% confidence intervals (CI) presented. Age and site were selected for adjustment of OR.

**Supplementary Table 2. Pregnancy outcomes of AMA patients for per-protocol analyses**

| **Outcome** | **ZYP group**  **(n=127)** | **Placebo group**  **(n=128)** | **Rate ratio in ZYP group (95% CI)** | **P** |
| --- | --- | --- | --- | --- |
| Implantation rate, no./total no. (%)^*^ | 88/274 (32.1%) | 65/275 (23.6%) | 1.36 (1.03-1.79) | 0.027 |
| Biochemical pregnancy | 77/127 (60.6%) | 59/128 (46.1%) | 1.32 (1.04-1.66) | 0.020 |
| Clinical pregnancy | 67/127 (52.8%) | 51/128 (39.8%) | 1.32 (1.01-1.73) | 0.039 |
| Live birth | 53 (41.7%) | 44 (34.4%) | 1.21 (0.89-1.66) | 0.226 |
| Singleton | 40 (31.5%) | 37 (28.9%) | 1.09 (0.75-1.58) | 0.652 |
| Twin | 13 (10.2%) | 7 (5.5%) | 1.87 (0.77-4.54) | 0.157 |
| Pregnancy loss, no./total no. (%) |  |  |  |  |
| Among biochemical pregnancy | 21/77 (27.3%) | 14/59 (23.7%) | 1.15 (0.64-2,06) | 0.639 |
| Among clinical pregnancy^†^ | 14/67 (20.9%) | 7/51 (13.7%) | 1.52 (0.66-3.50) | 0.313 |
| First trimester | 9/67 (13.4%) | 4/51 (7.8%) | 1.71 (0.56-5.26) | 0.337 |
| Second trimester | 3/67 (4.5%) | 3/51 (5.9%) | 0.76 (0.16-3.61) | 1.000 |
| Birth weight, g, median (IQR range) |  |  |  |  |
| Singleton | 3400 (3100-3575) | 3250 (2900-3638) |  | 0.312 |
| Twin | 2375 (1908-2775) | 2188 (1800-2338) |  | 0.062 |

Data are n (%), n/N (%) or median (IQR range) unless otherwise specified. P-values ＜ 0.05 were considered statistically significant.

ITT: intention-to-treat set; ZYP: Zishen Yutai Pill; CI: confidence interval; IQR: interquartile.

^*^Implantation rate = Number of gestational sacs/number of embryos transferred.

^†^In ZYP group, two cases of pregnancy loss occurred, but when pregnancy loss ocurred was unknown.

**Supplementary Table 3. Pregnancy outcomes of overweight/obese patients for per-protocol analyses**

| **Outcome** | **ZYP group**  **(n=134)** | **Placebo group**  **(n=121)** | **Rate ratio in ZYP group (95% CI)** | **P** |
| --- | --- | --- | --- | --- |
| Implantation rate, no./total no. (%)^*^ | 103/257 (40.1%) | 78/239 (32.6%) | 1.23 (0.97-1.56) | 0.085 |
| Biochemical pregnancy | 83/134 (61.9%) | 65/121 (53.7%) | 1.15 (0.93-1.43) | 0.184 |
| Clinical pregnancy | 75/134 (56.0%) | 61/121 (50.4%) | 1.11 (0.88-1.40) | 0.374 |
| Live birth^#^ | 63 (47.4%) | 50 (41.3%) | 1.15 (0.87-1.51) | 0.333 |
| Singleton | 40 (30.1%) | 40 (33.1%) | 0.91 (0.63-1.31) | 0.609 |
| Twin | 23 (17.3%) | 10 (8.3%) | 2.09 (1.04-4.22) | 0.033 |
| Pregnancy loss, no./total no. (%) |  |  |  |  |
| Among biochemical pregnancy | 16/82 (19.5%) | 13/65 (20.0%) | 0.98 (0.51-1.88) | 0.941 |
| Among clinical pregnancy^†^ | 11/75 (14.7%) | 11/61 (18.0%) | 0.81 (0.38-1.75) | 0.596 |
| First trimester | 5/75 (6.7%) | 8/61 (13.1%) | 0.51 (0.18-1.47) | 0.203 |
| Second trimester | 4/75 (5.3%) | 3/61 (4.9%) | 1.08 (0.25-4.65) | 1.000 |
| Birth weight, g, median (IQR range) |  |  |  |  |
| Singleton | 3520 (3150-3750) | 3500 (3100-3650) |  | 0.356 |
| Twin | 2600 (2288-2900) | 2500 (2250-2800) |  | 0.464 |

Data are n (%), n/N (%) or median (IQR range) unless otherwise specified.

ITT: intention-to-treat set; ZYP: Zishen Yutai Pill; CI: confidence interval; IQR: interquartile. P-values ＜ 0.05 were considered statistically significant.

^#^The denominators for live birth in the per-protocol set were 133 (ZYP group) and 121 (placebo group), respectively. One participant in the ZYP group underwent intervention as protocol required but was lost to follow-up, thus without live birth outcome record.

^*^Implantation rate = Number of gestational sacs/number of embryos transferred.

^†^In ZYP group, two cases of pregnancy loss occurred, but when pregnancy loss occurred were unknown.

**Supplementary Table 4. Hormone and oocyte indices among advanced maternal age (AMA, ≥35 yrs) and non-AMA (<35) population**

| **Characteristics** | **AMA group (n=427)** | **Non-AMA group (n=1838)** | **P Value** |
| --- | --- | --- | --- |
| AFC (n) | 12 (10-16) | 15 (11-20) | <0.001 |
| Basal hormone levels |  |  |  |
| E_2_ (pmol/L) | 139.7 (104.0-198.4) | 141.3 (106.4-191.4) | 0.685 |
| T (nmol/L) | 1.0 (0.7-1.4) | 1.2 (0.8-1.8) | <0.001 |
| FSH (international units/L) | 7.2 (6.1-8.3) | 6.6 (5.6-7.9) | <0.001 |
| LH (international units/L) | 4.3 (3.2-5.6) | 4.5 (3.3-6.1) | 0.007 |
| FSH/LH | 1.7 (1.3-2.3) | 1.5 (1.1-2.0) | <0.001 |
| Proportion of FSH/LH ≥3 | 40 (9.4) | 104 (5.7) | 0.005 |
| Oocytes retrieved, no. | 10 (7-14) | 13 (9-17) | <0.001 |
| Matured oocytes retrieved, no. | 8 (6-12) | 11 (8-14) | <0.001 |
| Cleavage embryos, no. | 7 (4-11) | 9 (6-13) | <0.001 |
| Fertilization embryos, no. | 8 (5-12) | 10 (6-13) | <0.001 |
| 2PN fertilization embryos, no. | 6 (4-9) | 8 (5-11) | <0.001 |
| Available embryo, no. | 4 (2-7) | 5 (3-8) | <0.001 |
| Good-quality embryos, no. | 3 (1-5) | 4 (1-7) | <0.001 |
| E2 on hCG trigger day (pmol/L) | 10437.5 (6829.9-15781.0) | 13457.9 (8648.4-25546.1) | <0.001 |
| Progesterone on hCG trigger day (mg/L) | 0.9 (0.7–1.2) | 1.0 (0.7–1.3) | 0.002 |

Data are n (%) or median (interquartile range).

AMA, advanced maternal age; yr, year; IVF, in vitro fertilization; BMI, body mass index; AFC, antral follicular count; E_2_, estradiol; T, testerone; FSH, follicular-stimulating hormone; LH, luteinizing hormone.

**Supplementary Table 5. Hormone and oocyte indices among overweight/obese and non- overweight/obese population**

| **Characteristics** | **Overweight/obese**  **group (n=468)** | **Non-overweight/obese**  **group (n=1797)** | **P Value** |
| --- | --- | --- | --- |
| AFC (n) | 15 (12-21) | 14 (11-19) | 0.008 |
| Basal sex hormone levels |  |  |  |
| E_2_ (pmol/L) | 127.2 (99.2-169.6) | 146.8 (109.8-198.2) | <0.001 |
| T (nmol/L) | 1.2 (0.8-1.7) | 1.2 (0.8-1.7) | 0.868 |
| FSH (international units/L) | 6.2 (5.4-7.3) | 6.9 (5.8-8.1) | <0.001 |
| LH (international units/L) | 4.1 (2.9-5.8) | 4.6 (3.4-6.1) | <0.001 |
| LH/FSH | 0.6 (0.5-0.9) | 0.7 (0.5-0.9) | 0.411 |
| Oocytes retrieved, no. | 12 (8-17) | 12 (8-17) | 0.181 |
| Matured oocytes retrieved, no. | 11 (8-15) | 10 (7-14) | 0.015 |
| Cleavage embryos, no. | 9 (6-12) | 9 (5-13) | 0.764 |
| Fertilization embryos, no. | 9 (6-13) | 9 (6-13) | 0.525 |
| 2PN fertilization embryos, no. | 8 (5-11) | 7 (4-11) | 0.829 |
| Available embryo, no. | 4 (3-7) | 5 (3-8) | 0.080 |
| Good-quality embryos, no. | 4 (2-6) | 3 (1-6) | 0.169 |
| E2 on hCG trigger day (pmol/L) | 11634.8 (7610.5-17142.6) | 13199.2 (8466.8-18115.1) | 0.003 |
| Progesterone on hCG trigger day (mg/L) | 0.9 (0.7–1.2) | 1.0 (0.7–1.3) | 0.005 |

Data are n (%) or median (interquartile range).

AMA, advanced maternal age; yr, year; IVF, in vitro fertilization; BMI, body mass index; AFC, antral follicular count; E_2_, estradiol; T, testerone; FSH, follicular-stimulating hormone; LH, luteinizing hormone.
